# Collective Almost Synchronisation in Complex Networks

**DOI:** 10.1371/journal.pone.0048118

**Published:** 2012-11-08

**Authors:** Murilo S. Baptista, Hai-Peng Ren, Johen C. M. Swarts, Rodrigo Carareto, Henk Nijmeijer, Celso Grebogi

**Affiliations:** 1 Institute for Complex Systems and Mathematical Biology, University of Aberdeen, SUPA, Aberdeen, United Kingdom; 2 Department of Information and Control Engineering, Xi'an University of Technology, Xi'an, China; 3 Department of Mechanical Engineering, Dynamics and Control Group, Eindhoven University of Technology, Eindhoven, The Netherlands; 4 Escola Politecnica, Universidade de São Paulo, São Paulo, Brazil; University of Maribor, Slovenia

## Abstract

This work introduces the phenomenon of Collective Almost Synchronisation (CAS), which describes a universal way of how patterns can appear in complex networks for small coupling strengths. The CAS phenomenon appears due to the existence of an approximately constant local mean field and is characterised by having nodes with trajectories evolving around periodic stable orbits. Common notion based on statistical knowledge would lead one to interpret the appearance of a local constant mean field as a consequence of the fact that the behaviour of each node is not correlated to the behaviours of the others. Contrary to this common notion, we show that various well known weaker forms of synchronisation (almost, time-lag, phase synchronisation, and generalised synchronisation) appear as a result of the onset of an almost constant local mean field. If the memory is formed in a brain by minimising the coupling strength among neurons and maximising the number of possible patterns, then the CAS phenomenon is a plausible explanation for it.

## Introduction

Spontaneous emergence of collective behaviour is common in nature [1]–[3]. It is a natural phenomenon characterised by a group of individuals that are connected in a network by following a dynamical trajectory that is different from the dynamics of their own. Since the work of Kuramoto [4], the spontaneous emergence of collective behaviour in networks of phase oscillators with full bidirectionally connected nodes or with nodes connected by some special topologies [5] is analytically well understood. Kuramoto considered a fully connected network of an infinite number of phase oscillators. If 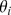 is the variable describing the phase of an oscillator 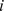 in the network, and 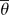 represents the mean field defined as 
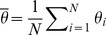
, collective behaviour appears in the network because every node becomes coupled to the mean field. Peculiar characteristics of this collective behaviour is that not only 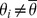 but also nodes evolve in a way that cannot be described by the evolution of only one individual node, when isolated from the network.

In contrast to collective behaviour, another widely studied behaviour of a network is when all nodes behave equally, and their evolution can be described by an individual node when isolated from the network. This state is known as complete synchronisation [6]. If 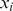 represents a scalar state variable of an arbitrary node 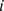 of the network and 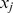 of another node 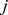, and 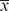 represents the mean field of a network calculated with this scalar state variable, complete synchronisation appears when 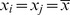, for all time. The main mechanisms responsible for the onset of complete synchronisation in dynamical networks were clarified in [7]–[9]. In networks whose nodes are coupled by non-linear functions, such as those that depend on time-delays [9] or those that describe how neurons chemically connect [10], the evolution of the synchronous nodes might be different from the evolution of an individual node, when isolated from the network. However, when complete synchronisation is achieved in such networks, 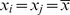.

In natural networks as biological, social, metabolic, neural networks, etc, [11], the number of nodes is often large but finite; the network is not fully connected and heterogeneous. The later means that each node has a different dynamical description or the coupling strengths are not all equal for every pair of nodes, and one will not find two nodes, say it 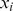 and 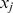, that have equal trajectories. For such heterogeneous networks, as in [12]–[14], found in natural networks and in experiments [15], one expects to find other weaker forms of synchronous behaviour, such as practical synchronisation [16], phase synchronisation [15], time-lag synchronisation [17], generalised synchronisation [18].

We report a phenomenon that may appear in complex networks “far away” from coupling strengths that typically produce complete synchronisation or these weaker forms of synchronisation. However, the reported phenomenon can be characterised by the same conditions used to verify the existence of these weaker forms of synchronisation. We call it Collective Almost Synchronisation (CAS). It is a consequence of the appearance of an approximately constant local mean field and is characterised by having nodes with trajectories evolving around stable periodic orbits, denoted by 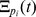, and regarded as a CAS pattern. The appearance of an almost constant mean field is associated with a regime of weak interaction (weak coupling strength) in which nodes behave independently [19], [20]. In such conditions, even weaker forms of synchronisation are ruled out to exist. But, contrary to common notion based on basic statistical arguments, we show that actually it is the existence of an approximately constant local mean field that paves the way for weaker forms of synchronisation (such as almost [16], time-lag, phase, or generalised synchronisation) to occur in complex networks.

Denote all the 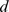 variables of a node 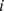 by 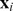, then we define that this node presents CAS if the following inequality
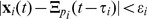
(1)is satisfied for *all the time*. The double vertical bar 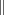 represents that we are taking the absolute difference between vector components appearing inside the bars (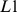 norm). However, this equation could be rewritten in terms of each vector component. 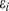 is a small quantity, not arbitrarily small, but reasonably smaller than the envelop of the oscillations of the variables 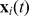. Its magnitude depends on the variance around the local mean field of node 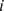. 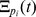 is the 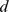-dimensional CAS pattern. It is determined by the effective coupling strength 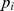, a quantity that measures the influence on the node 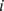 of the nodes that are connected to it, and the expected value of the local mean field at the node 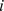, denoted by 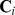. The local mean field, denoted by 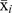, is defined only by the nodes that are connected to the node 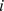. The CAS pattern is the solution of a simplified set of equations describing the network when 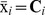. According to Eq. (1), if a node in the network presents the CAS pattern, its trajectory stays intermittently close to the CAS pattern but with a time-lag between the trajectories of the node and of the CAS pattern. This property of the CAS phenomenon shares similarities with the way complete synchronisation appears in networks of nodes coupled under time-delay functions [9]. In such networks, nodes become completely synchronous to a solution of the network that is different from the solution of an isolated node of the network. Additionally, the trajectory of the nodes present a time-lag to this solution.

The CAS phenomenon inherits the three main characteristics of a collective behaviour: (a) the variables of a node 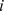 (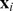) differ from both the mean field 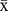 and the local mean field 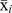; (b) if the local mean fields of a group of nodes and their effective coupling are either equal or approximately equal, that causes all the nodes in this group to follow the same or similar behaviours; (c) there can exist an infinitely large number of different behaviours (CAS patterns).

There is a wide belief in the academic community that patterns appearing in a complex network due to a collective behaviour cannot exist if nodes interact by extremely weak couplings. Contrary to this line of thinking, in Refs. [21]–[23], was shown that quantities that measure the level of collective behaviour in networks can be far from zero even when the coupling strength among nodes is small. This work shows that in fact there exists an enormous amount of patterns in such networks, infinitely many if the network has infinite nodes. These patterns were probably not observed before because not only they appear in a large number but also similar patterns appear with a time-lag, a characteristic that endows the network with its stochastic behaviour. This stochastic behaviour allows us to use the Central Limit Theorem to explain why the local mean field defined in the observable variable 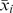 is approximately constant. Consequently, it is possible to arrive at an approximate equation for every node of the network as if they were detached from it. This framework of dealing with the network effect as a local mean field and applying the Central Limit approach has been proposed in Ref. [24] to show that the local mean field defined by the coupling term was shown to be approximately zero when the phenomenon of hub synchronisation appears.

As examples of how common this phenomenon could be, we have asserted its appearance in heterogenous networks of nodes coupled diffusively in the thermodynamic limit, in large networks of chaotic maps, Hindmarsh-Rose neurons, and Kuramoto oscillators, and finally in systems that are models for the appearance of collective motion in social, economical, and animal behaviour. In addition, we have performed a series of numerical experiments in these systems to support our claims.

## Methods

### The CAS phenomenon

Consider a network of 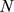 nodes described by
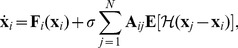
(2)where 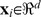 is a d-dimensional vector describing the state variables of the node 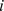, 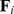 is a 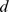-dimensional vector function representing the dynamical system of the node 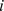, 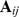 is the adjacent connection matrix, 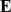 is the coupling function as defined in [7], 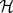 is an arbitrary differentiable transformation. The degree of a node can be calculated by 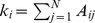. Assume in the following that 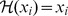. To extend the analysis to a nonlinear function 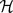, see the results for the Kuramoto network (Eq. (11)). In such a case, we need to rewrite the coupling term in Eq. (2) as a function of the local mean field.

The CAS phenomenon appears when the local mean field of a node 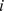, defined as
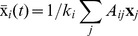
(3)is approximately constant and
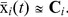
(4)


Then, the equations for the network can be described in terms of the local mean field by
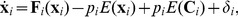
(5)where 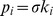 and the residual term is 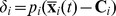. The CAS pattern of the node 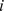 (a stable periodic orbit) is calculated in the variables that produce a finite bounded local average field. If all components of 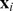 are bounded, then the CAS pattern is given by a solution of
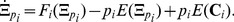
(6)which is just the same set of equations (5) without the residual term. So, if 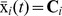, the residual term 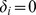, and if Eq. (6) has no positive Lyapunov exponents (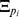 is a stable periodic orbit), then the node 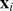 describes a stable periodic orbit. If 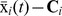 is larger than zero but 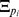 is a stable periodic orbit, then the node 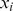 describes a perturbed version of 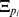.

Notice from Eq. (6) that for 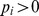, the CAS pattern will not be described by 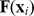 and therefore does not belong to the synchronisation manifold. On the other hand, 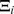 is induced by the local mean field as typically happens in synchronous phenomenon due to collective behaviour. This property of the CAS phenomenon shares similarities with the way complete synchronisation appears in networks of nodes coupled under time-delay functions [9]. In such networks, nodes become completely synchronous to a solution of the network that is different from the solution of an isolated node of the network. Additionally, the trajectory of the nodes present a time-lag to this solution, as shown in Eq. (1).

To understand the reason why the CAS phenomenon appears when 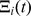 is a sufficiently stable periodic orbit, we study the variational equation of the CAS pattern (6)
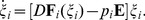
(7)obtained by linearising Eq. (6) around 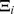 by making 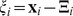. This equation is assumed to produce no positive Lyapunov exponents. We also assume here that the Lyapunov exponents are regular [25], meaning that perturbations do not destroy the periodic orbit. Therefore, small fluctuations of the local mean field do not cause the trajectory to scape the neighbourhood of the CAS pattern. As a consequence, neglecting the existence of the time-lag between 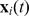 and 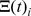, the trajectory of the node 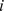 oscillates about 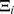, and 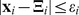, for all the time, satisfying Eq. (1), where 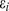 depends on the variance of the local mean field and also on 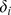. If there are two nodes 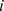 and 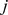, which feel similar local mean fields and 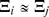 (so, 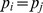), then 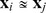, for all the time.

To understand why the nodes that present CAS have also between them a time-lag type of synchronisation, notice that there is a transient time in order for Eq. (6) to describe well in an approximate sense the solutions of Eq. (5), if we consider a typical situation where initial conditions are not equal and are not placed along the asymptotic limiting set of the CAS pattern. At the time the trajectory of all nodes approach their CAS pattern, even two nodes 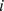 and 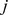 that have identical CAS patterns (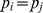 and 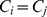) have trajectories that arrive in different places of 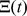. The CAS pattern is a stable periodic orbit and we obtain it by considering in Eq. (6) an arbitrary initial condition. Therefore, the asymptotic trajectory obtained in Eq. (5) will be typically in a different place than the asymptotic trajectory obtained in Eq. (6). This fact has taken us to include the time-delay 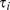 in Eq. (1) in order to have an equation that can be used in typical experimental situations. When dealing with numerical experiments, the time-delay 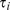 could be removed from Eq. (1) by resetting the integration time for the CAS pattern after the trajectory of the node arrives to its neighbourhood. But this would only be possible when we have access to the integration time. As a result, nodes that are collectively almost synchronous obey Eq. (1). In addition, two nodes that present CAS have also a time-lag between their trajectories for the same reason.

If the network has unbounded state variables (as it is the case of Kuramoto networks [4]), the CAS pattern is the periodic orbit of period 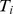 defined in the velocity space such that 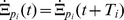.

Notice that whereas Eqs. (2) and (5) represent a 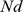-dimensional system, Eq. (6) has only dimension 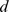.

The existence of this approximately constant local mean field is a consequence of the Central Limit Theorem, applied to variables with correlation (for more details, see the following section). The expected value of the local mean field can be calculated by
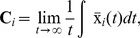
(8)where in practice we consider 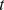 to be large, but finite. The larger the degree of a node, the higher is the probability for the local mean field to be close to an expected value and smaller its variance. If the probability to find a certain value for the local mean field of the node 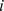 does not depend on the higher order moments of 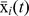, then this probability tends to be Gaussian for sufficiently large 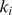. As a consequence, the variance 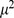 of the local mean field is proportional to 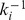.

There are two criteria for the node 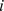 to present the CAS phenomenon:

#### Criterion 1

The Central Limit Theorem can be applied, i.e., 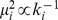. Therefore, the larger the degree of a node, the smaller the variation of the local mean field 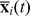 about its expected value 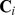.

#### Criterion 2

The CAS pattern 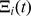 describes a stable periodic orbit exponentially and uniformly attractive, such that the perturbation 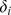 in Eq. (5) does not take the trajectory of the node 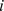 away from its CAS pattern. The node trajectory can be considered to be a perturbed version of its CAS pattern. The more stable the faster trajectories of nodes come to the neighbourhood of the periodic orbits (CAS patterns).

Whenever the Central Limit Theorem applies, the random variables involved are independent. But, the Central Limit Theorem can also be applied to variables with correlation. If nodes that present the CAS phenomenon are locked to the same CAS pattern, their trajectories still arrive to the CAS pattern at different “random” times, allowing for the Central Limit Theorem to be applied. Assume a time when all nodes reach their asymptotic state and the nodes that present the CAS pattern have trajectories that are close to their CAS pattern. Imagine a group of nodes that have the same CAS pattern. Their trajectories can be approximately described by 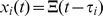, where 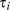 represents what we call “random” times, meaning that for every two nodes 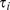 and 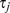 are decorrelated.

The time-lag between two nodes (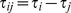) is approximately constant, since the CAS pattern has a well defined period, and the trajectories of these nodes are locked into it.

The CAS phenomenon exists when a node has an approximately constant local mean field and its CAS pattern is a stable periodic orbit. If the equation for the CAS pattern (Eq. (6)) presents coexistence of attractors, nodes will still be in a CAS state if the CAS conditions are satisfied. In our simulations, the range of initial conditions that have trajectories that go asymptotically to the same stable periodic orbit is large. Likely because the CAS pattern equation has global stable attractors for the parameters we have studied numerically.

## Results

This session is dedicated to illustrate and explain the appearance of the CAS phenomenon in 5 different systems.

### CAS in heterogeneous and homogeneous networks of nodes coupled diffusively in the thermodynamic limit (infinite nodes fully connected)

The equations for a heterogenous network of nodes coupled diffusively can be described by
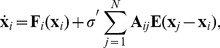
(9)where we have renormalised 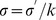.

In the thermodynamics limit, when 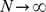 and 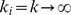 (fully connected network), the network can be imagined as describing a discretised spatially coupled network. If the renormalised coupling is sufficiently small such that the central limit theorem can be applied, the expected value of the local mean field is constant and equals 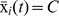, for all 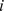.

Rewriting Eq. (9) as a function of the expected value of the local mean field, we arrive at
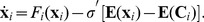
(10)The CAS pattern 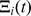 of a node 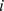 is also described by Eq. (10). Every node 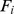 that describes a stable periodic orbit is in a CAS state. Every two nodes that are in a CAS state are phase-locked and will present other types of weak synchronisation.

If the network is homogeneous and the dynamics of every node is described by the same function 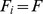, then every node will be described by the same set of 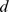-dimensional ODEs. We assume that initial conditions are not identical. If Eq. (10) describes a stable periodic orbit, then every node's trajectory is described by the same stable periodic orbit, the CAS pattern. That will result in a network that has no positive Lyapunov exponents, but because of the time-lag among the node's equal periodic trajectories, the network will appear not to present patterns due to collective behaviours, because the nodes will be out of phase with respect to each other.

#### On the constancy of expected value of the local mean field with respect to a varying 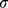


The expected value will depend on 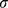, but for every value of 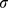, all the nodes will present the same constant expected value of the local mean field 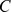. If the alteration in 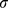 does not produce positive Lyapunov exponents in Eq. (10) for every two nodes, then the existence of the CAS phenomenon for these two nodes is not destroyed, if 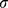 is altered.

### CAS in a network of coupled maps

As another example to illustrate how the CAS phenomenon appears in a complex network, we consider a network of maps whose node dynamics is described by 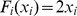 mod(1). The network composed, say, by 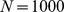 maps, is represented by 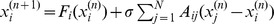 mod(1), where the upper index 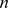 represents the discrete iteration time, and 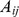 is the adjacency matrix of a scaling-free network. The degree distribution of the scaling-free networks considered in this work follow a power law with coefficient close to −2.621.

**Figure 1:**
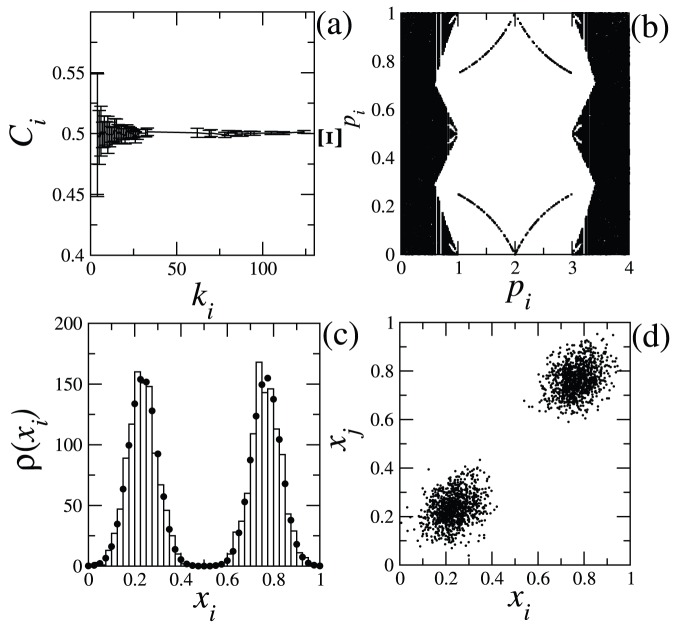
Results for a network of coupled maps. (a) Expected value of the local mean field of the node 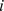 against the node degree 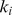. The error bar indicates the variance (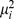) of 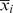. (b) A bifurcation diagram of the CAS pattern [Eq. (6)] considering 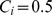. (c) Probability density function of the trajectory of a node with degree 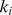 = 80 (therefore, 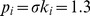, 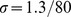). (d) A return plot considering two nodes (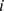 and 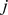) with the same degree 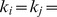
80.

The map has a constant probability density. When such a map is connected in a network, the probability measure of the trajectory is no longer constant, but still symmetric and having an average value of 0.5. As a consequence, nodes that have a sufficient amount of connections (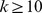) feel a local mean field, say, within 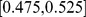, (deviating of 5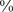 about 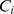 = 0.5) and 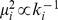 (**criterion 1**), as shown in Fig. 1(a). Therefore, such nodes have propensity to present the CAS phenomenon. In (b) we show a bifurcation diagram of the CAS pattern, 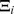, obtained from Eq. (6) by using 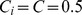, as we vary 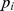. Nodes in this network that have propensity to present the CAS phenomenon will present it if additionally 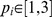; the CAS pattern is described by a period-2 stable orbit (**criterion 2**). This interval can be calculated by solving 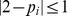. In (c) we show the probability density function of the trajectory of a node that present the CAS phenomenon. The density is centred at the position of the period-2 orbit of the CAS pattern and for most of the time Eq. (1) is satisfied. The filled circles are fittings assuming that the probability density is given by a Gaussian distribution. Therefore, there is a high probability that 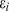 in Eq. (1) is small. In (d) we show a plot of the trajectories of two nodes that have the same degree which is equal to 80. We chose nodes which present no time-lag between their trajectories and the trajectory of the pattern. If there was a time-lag, the points in (d) would not be only aligned along the diagonal (identity) line, but they would also appear off-diagonal.

#### On the constancy of expected value of the local mean field with respect to a varying 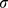


The expected value for the local mean field for all the nodes is constant, 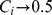 (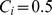, in the thermodynamic limit), and does not depend on the coupling strength 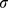. That is a consequence of the symmetrical properties of the probability measure of the trajectory. Therefore, changes in the coupling strength do not alter 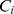. If 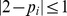, the CAS state of the nodes is maintained and the synchronous phenomena observed in the network might be maintained as well, if 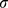 is altered.

### CAS in the Kuramoto network

An illustration of this phenomenon in a network composed by nodes having heterogeneous dynamical descriptions and a nonlinear coupling function is presented in a random network of 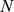 = 1000 Kuramoto oscillators. This network was constructed such that the average degree is 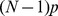, where 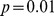 is the probability of each two nodes to be connected. This probability is slightly larger than 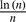, resulting in a network that is almost surely connected.

We rewrite the Kuramoto network model in terms of the local mean field, 
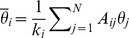
. Using the coordinate transformation 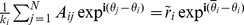, the dynamics of node 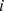 is described by
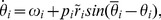
(11)where 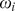 is the natural frequency of the node 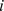, taken from a Gaussian distribution centred at zero and with standard deviation of 4. If 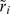 = 1, all nodes coupled to node 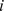 are completely synchronous with it. If 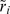 = 0, there is no synchronisation between the nodes that are coupled to the node 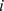. Since the phase is an unbounded variable, the CAS phenomenon should be verified by the existence of an approximate constant local mean field in the frequency variable 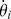. If 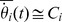, which means that 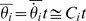, then Eq. (11) describes a periodic orbit (the CAS pattern), regardless the values of 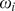, 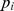, and 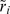, since it is an autonomous two-dimensional system; chaos cannot exist. Therefore, **criterion 2** is always satisfied in a network of Kuramoto oscillators. We have numerically verified that **criterion 1** is satisfied for this network for 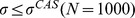, where 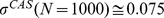. Complete synchronisation is achieved in this network for 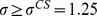. So, the CAS phenomenon is observed for a coupling strength that is 15 times smaller than the one that produces complete synchronisation.

**Figure 2:**
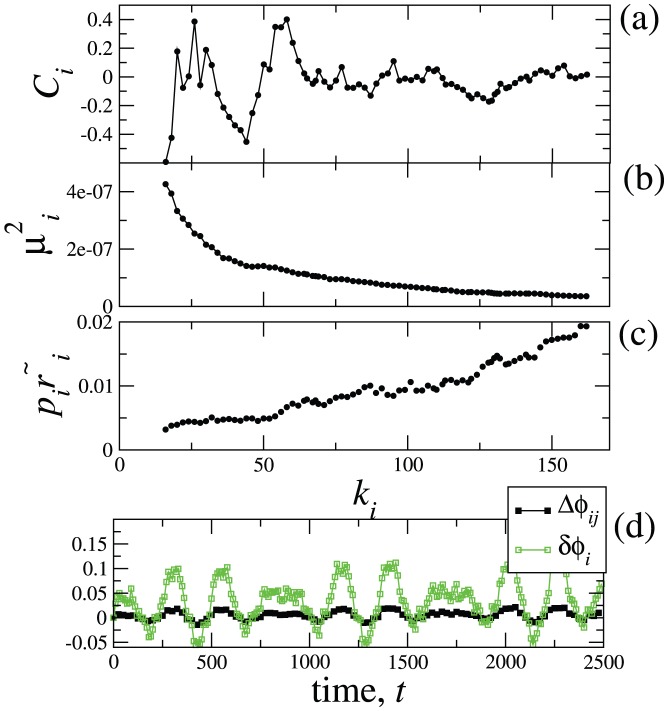
Results for the Kuramoto network. Results for 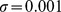. (a) Expected value of the local mean field 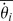 of a node with degree 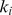, picked randomly. Nodes with the same degree present nearly identical local mean fields. (b) The variance 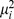 of the local mean field. (c) Relationship between the value of 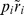 and 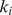. (d) Phase difference 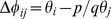 between two nodes, one with degree 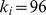 and the other with degree 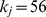; the phase difference 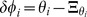 between the phases of the trajectory of the node 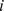 with degree 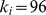 and the phase of its CAS pattern.

For the following results, we choose 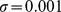. Since the natural frequencies have a distribution centred at zero, it is expected that, for nodes with higher degrees, the local mean field is close to zero (see Fig. 2(a)). In (b), we show the variance of the local mean field of the nodes with degree 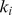. The fitting produces 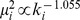 (**criterion 1**). In (c), we show the relationship between the value of 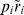 and the value of the degree 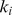. In order to calculate the CAS pattern of a node with degree 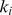, we need to use the value of 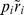 (which is obtained from this figure) and the measured 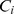 as an input in Eq. (11). We pick two arbitrary nodes, 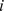 and 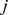, with degrees 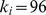 and 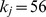, respectively, with natural frequencies 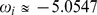 and 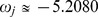. In (d), we show that phase synchronisation is verified between these two nodes assuming that 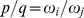. We also show the phase difference 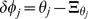 between the phases of the trajectory of the node 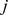 with degree 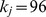 and the phase of its CAS pattern, for a time interval corresponding to approximately 2500/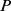 cycles, where the period of the cycles in node 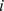 is calculated by 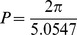. Phase synchronisation between nodes 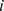 and 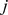 is a consequence of the fact that the phase difference between the nodes and their CAS patterns is bounded.

Phase synchronisation will be rational and stable whenever nodes with different natural frequencies 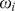 become locked to Arnold tongues [26], [27] induced by the coupling 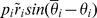. Notice that whereas the instantaneous frequency of oscillation of a node isolated from the network (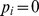) is given by its natural frequency 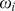 of rotation (which can be an irrational number), a node 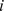 that is in CAS has an instantaneous frequency that is given by 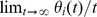, assumed to be a rational rotation and for that reason typically differs from 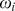. Irrational phase synchronisation will appear between two nodes in this network if we allow the CAS pattern to be described by a quasi-periodic rotation.

#### On the constancy of expected value of the local mean field with respect to a varying 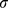


In the thermodynamic limit, when a fully connected network has an infinite number of nodes, 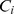 does not change as one changes the coupling 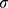, since it only depends on the mean field of the frequency variable (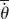). As a consequence, if there is the CAS phenomenon and phase synchronisation between two nodes with a ratio of 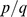 for a given value of 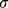, changing 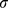 does not change the ratio 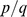. Therefore phase synchronisation is stable under alterations in 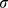.

### CAS in a network of Hindmarsh-Rose neurons

As an example to illustration how the CAS phenomenon appears in a complex network, we consider a scaling-free network formed by, say, 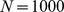 Hindmarsh-Rose neurons, with neurons coupled electrically. The network is described by
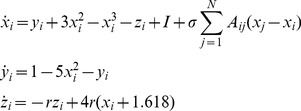
(12)where 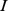 = 3.25 and 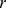 = 0.005. The first coordinate of the equations that describe the CAS pattern is given by
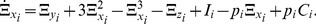
(13)


**Figure 3:**
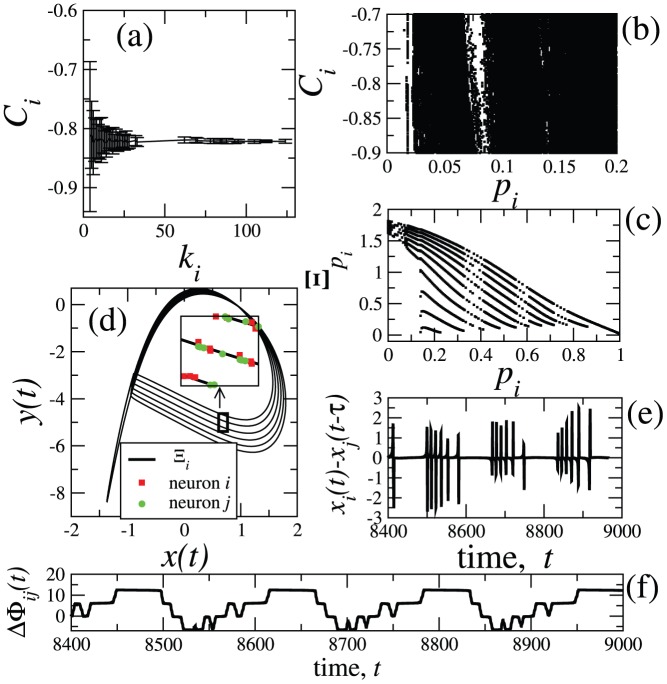
Results for a network of Hindmarsh-Rose neurons. (a) Expected value of the local mean field of the node 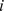 against the node degree 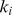. The error bar indicates the variance (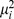) of 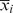. (b) Black points indicate the value of 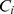 and 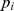 for Eq. (13) to present a stable periodic orbit (no positive Lyapunov exponents). The maximal values of the periodic orbits obtained from Eq. (13) is shown in the bifurcation diagram in (c) considering 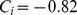 and 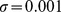. (d) The CAS pattern for a neuron 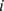 with degree 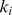 = 25 (with 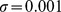 and 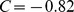). In the inset, the same CAS pattern of the neuron 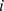 and some sampled points of the trajectory for the neuron 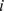 and another neuron 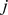 with degree 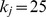. (e) The difference between the first coordinates of the trajectories of neurons 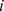 and 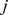, with a time-lag of 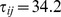. (f) Phase difference between the phases of the trajectories for neurons 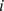 and 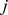.

The others are given by 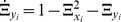, 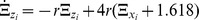. In this network, we have numerically verified that **criterion 1** is satisfied for neurons that have degrees 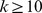 if 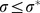, with 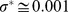. In Fig. 3(a), we show the expected value 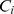 of the local mean field of the first coordinate 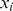 of a neuron 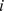 with respect to the neuron degree (indicated in the horizontal axis), for 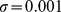. The error bar indicates the variance of 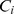 which fits to 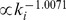. In Fig. 3(b), we show a parameter space to demonstrate that the CAS phenomenon is a robust and stable phenomenon. Numerical integration of Eqs. (12) for 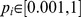 produces 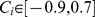. We integrate Eq. (13) by using 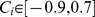 and 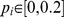, to show that the CAS pattern is stable for most of the values. So, variations in 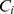 of a network caused by changes in a parameter do not modify the stability of the CAS pattern calculated by Eq. (13). For 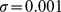, Eqs. (12) yields many nodes for which 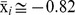. So, to calculate the CAS pattern for these nodes, we use 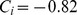 and 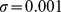 in Eqs. (13). The CAS pattern obtained, as we vary 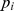, is shown in the bifurcation diagram in Fig. 3(c), by plotting the local maximal points of the CAS patterns. **Criterion 2** is satisfied for most of the range of values of 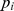 that produces a stable periodic CAS pattern. A neuron that has a degree 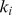 is locked to the CAS pattern calculated by integrating Eqs. (13) using 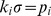 and the measured expected value for the local mean field, 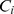. In Fig. 3(d), we show the periodic orbit corresponding to a CAS pattern associated to a neuron 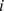 with degree 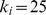 (for 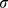 = 0.001) and in the inset the sampled points of the trajectories of this same neuron 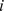 and of another neuron 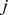 that has not only equal degree (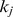 = 25), but it feels also a local mean field of 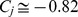. In Fig. 3(e), we show that these two neurons have a typical time-lag synchronous behavior. In Fig. 3(f), we observe 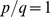 phase synchronisation between these two neurons for a long time, considering that the phase difference remains bounded by 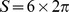 as defined in Eq. (17), where the number 6 is the number of spikings within one period of the slower time-scale. In order to verify Eq. (17) for all time, we need to choose a ratio that is approximately equal to 1 (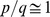), but not exactly 1 to account for slight differences in the local mean field of these two neurons. Phase was measured by integrating the differential phase equation proposed in the work of Ref. [28] that measures the amount of rotation of the tangent vector.

Since 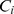 depends on 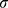 for networks that have neurons possessing a finite degree, we do not expect to observe a stable phase synchronisation in this network. Small changes in 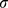 may cause small changes in the ratio 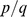. Notice however that Eq. (17) might be satisfied for a very long time, for 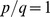. If neurons are locked to different CAS patterns (and therefore have different local mean field), Eqs. (1) and (17) are both satisfied, but phase synchronisation will not be 1∶1, but with a ratio of 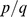 (see Sec. E in Supplementary Information for an example).

If neurons in this scaling-free network become completely synchronous, it is necessary that 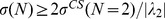 (Ref. [7]). 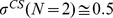 represents the value of the coupling strength when two bidirectionally coupled neurons become completely synchronous. 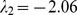 is the largest non-positive eigenvalue of the Laplacian matrix defined as 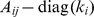. So, 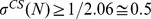. The CAS phenomenon appears when 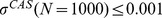, a coupling strength 500 times smaller than the one which produces complete synchronisation. Similar conclusions would be obtained when one considers networks of different sizes, with nodes having the same dynamical descriptions and same connecting topology.

#### On the constancy of expected value of the local mean field with respect to a varying 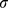


We have numerically verified that changes in the coupling strength 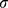 only slightly alter the value of the local mean field 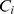. Therefore, we expect that the synchronous phenomena observed for a particular value of the coupling strength can be maintained by an alteration of 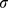, if the CAS is present in the network.

### CAS in systems of driven particles

The CAS phenomenon can also appear in a system of driven particles [29] that is a simple but powerful model for the onset of pattern formation in population dynamics [2], economical systems [30] and social systems [3]. In the work of Ref. [29], it was assumed that individual particles were moving at a constant speed but with an orientation that depends on the local mean field of the orientation of the individual particles within a local neighbourhood and under the effect of additional external noise. Writing an equivalent time-continuous description of the Vicsek particle model [29], the equations of motion for the direction of movement of a particle 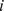, can be written as
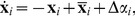
(14)where 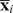 represents the local mean field of the orientation of the particle 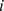 within a local neighbourhood and 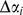 represents a small noise term. When 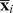 is approximately constant, the CAS pattern is described by a solution of 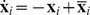, which will be a stable steady state (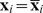) as long as 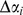 is sufficiently small. From the Central Limit Theorem, 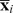 will be approximately constant as long as the neighbourhood considered is sufficiently large or the density of particles is sufficiently large.

## Analysis

### CAS and other weaker forms of synchronisation

If the CAS phenomenon is present in a network, other weaker forms of synchronisation can be detected. This link is fundamental when making measurements to detect the CAS phenomenon.

In Ref. [16], the phenomenon of almost synchronisation is introduced, when a master and a slave in a master-slave system of coupled oscillators have equal phases but their amplitudes can be different. If a node 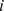 presents the CAS phenomenon [satisfying Eq. (1)] and 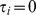 in Eq. (1), then the node 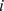 is almost synchronous to the pattern 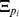.

Time-lag synchronisation [17] is a phenomenon that describes two identical signals, but whose variables have a time-lag with respect to each other, i.e. 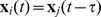. In practice, however, an equality between 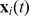 and 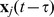 should not be expected to be typically found, but rather
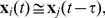
(15)meaning that there is not a constant 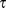 that can be found such that 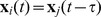. Another suitable way of writing Eq. (15) is by 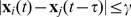. If two nodes 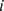 and 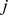 that present the CAS phenomenon, have the same CAS pattern, and 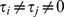, then
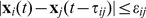
(16)or alternatively 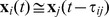, for most of the time, 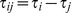 representing the time-lag between 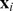 and 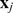. Equation (16) is satisfied for all the time, when the network is composed by elements that have only one time-scale, such as the Kuramoto oscillator. In neural networks whose neurons have more than one time-scale the delay 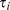 as well as 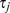 vary in time and therefore we do not find a 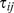 such that Eq. (16) is satisfied for all the time. This means that almost time-lag synchronisation occurs for two nodes that present the CAS phenomenon and that are almost locked to the same CAS pattern. Even though nodes that have equal or similar local mean field (which usually happens for nodes that have equal or similar degrees) become synchronous with the same CAS pattern (a stable periodic orbit), the value of their trajectories at a given time might be different, since their trajectories reach the neighbourhood of their CAS patterns in different places of the orbit. As a consequence, we expect that two nodes that exhibit the same CAS should present between themselves a time-lag synchronous behavior. For some small amounts of time, the difference 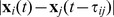 can be large, since 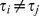 and 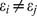, in Eq. (1). The closer 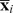 and 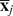 are to 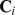, the smaller is 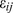 in Eq. (16).

Phase synchronisation [15] is a phenomenon where the phase difference, denoted by 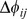, between the phases of two signals (or nodes in a network), 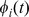 and 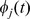, remains bounded for all time
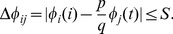
(17)In Ref. [15] 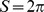 and 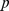 and 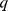 are two rational numbers. If 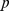 and 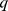 are irrational numbers and 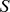 is a reasonably small constant, then phase synchronisation can be referred as to irrational phase synchronisation [31]. The value of 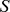 is calculated in order to encompass oscillatory systems that possess either a time varying time-scale or a variable time-lag. Simply make the constant 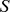 to represent the growth of the phase in the faster time scale during one period of the slower time scale. Phase synchronisation between two coupled chaotic oscillators was explained as being the result of a state where the two oscillators have all their unstable periodic orbits phase-locked [15]. Nodes that present the CAS phenomenon have unstable periodic orbits that have periods that are approximately given by multiples of the period of the stable periodic orbits described by 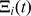. If 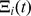 has a period 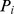 and the phase of this CAS pattern changes 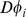 within one period, so the angular frequency is 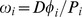. If 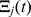 has a period 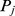 and the phase of its CAS patter changes 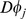 within one period, so the angular frequency is 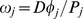. Then, the CAS patterns of these nodes are phase synchronous by a ratio of 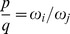. Since the trajectories of these nodes are bounded to these patterns, the nodes are phase synchronous by this same ratio, which can be rational or irrational. If two nodes 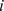 and 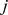 have the same CAS pattern, making observations in one node once every time another node crosses a Poincaré section results in a discrete set of points that are localised in the subspace of the nodes whose observations are being made. Such a localised set was demonstrated in [28] to be a direct consequence of phase synchronisation.

Assume additionally that, as one changes the coupling strengths between the nodes, the expected value 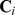 of the local mean field of a group of nodes remains the same. As a consequence, as one changes the coupling strengths, both the CAS pattern and the ratio 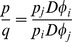 remain unaltered, and the observed phase synchronisation between nodes in this group is stable under parameter alterations.

In Ref. [24], synchronisation was defined in terms of the node 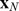 that has the largest number of connections, when 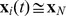 (which is equivalent to stating that 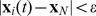), where 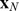 is assumed to be very close to the synchronization manifold 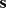 defined by 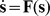. This type of synchronous behaviour was shown to exist in scaling free networks whose nodes have equal dynamics and that are linearly connected. This was called hub synchronisation.

The link between the CAS phenomenon with the hub synchronisation phenomenon [24], and generalised synchronisation can be explained as in the following. It is not required for nodes that present the CAS phenomenon for their error dynamics 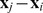 to be small. But for the following comparison, assume that 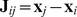 is small so that we can linearise Eq. (2) about another node 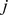. Assume also that 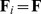. The variational equations of the error dynamics between two nodes 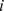 and 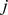 that have equal degrees are described by
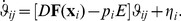
(18)In Ref. [24], hub synchronisation exists if Eq. (18), neglecting the coupling term 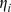, has no positive Lyapunov exponents. That is another way of stating that hub synchronisation between 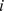 and 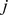 occurs when the variational equations of the modified dynamics 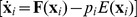 presents no positive Lyapunov exponent. In other words, in order to have hub synchronisation it is necessary that the modified dynamics of both nodes be describable by stable periodic oscillations. Hub synchronisation is the result of a weak form of generalised synchronisation, defined in terms of the linear stability of the error dynamics between two highly connected nodes. Unlike generalised synchronisation, hub synchronisation offers a way to predict, in an approximate sense, the trajectory of the synchronous nodes.

In contrast, the CAS phenomenon appears when the CAS pattern, which is different from the solution of the modified dynamics, becomes periodic. Another difference between the CAS and the hub synchronisation phenomenon is that whereas 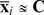 in the CAS phenomenon, 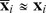 in the hub synchronisation, in order for 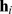 to be very small, and 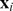 to be close to the synchronisation manifold. So, whereas hub synchronisation can be interpreted as being a type of practical synchronisation [16], CAS is a type of almost synchronisation.

In the work of Refs. [32], [33], it was numerically reported a new desynchronous phenomenon in complex networks. The network has no positive Lyapunov exponents but it presents a desynchronous non-trivial collective behaviour. A possible situation for the phenomenon to appear is when 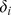 and 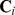 in Eq. (5) are either zero or sufficiently small such that the stability of the network is completely determined by Eq. (7), and this equation produces no positive Lyapunov exponent. Assume now that 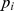 in Eq. (6) is appropriately adjusted such that the CAS pattern for every node 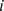 is a stable periodic orbit. The variational Eqs. (7) for all nodes have no positive Lyapunov exponents. If additionally, 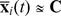, then the network in Eq. (5) possesses no positive Lyapunov exponent. Therefore, networks that present the CAS phenomenon for all nodes might present the desynchronous phenomenon reported in Refs. [32], [33]. The CAS phenomenon becomes different from the phenomenon of Refs. [32], [33] if for at least one node, Eq. (6) produces a chaotic orbit.

In the works of Refs. [34], [35] it was reported the phenomenon of explosive synchronisation in networks of oscillators whose natural frequency is correlated to its degree. This phenomenon is characterised by the abrupt appearance of synchronisation when the coupling strength among the nodes is varied. For a large range of small values of the coupling strength, the level of synchrony (measured by the order parameter or phase synchronisation) remains small. It abruptly increases following a typical first-order transition at some critical coupling. This suggests that in such networks the CAS phenomenon can appear for a large range of the coupling strengths, the same range that produces a low level of synchrony in the network.

### CAS and generalised synchronisation

Generalised synchronisation [18], [36] is a common behaviour in complex networks [37]–[39], and should be expected to be found typically. This phenomenon is defined as 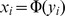, where 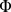 is considered to be a continuous function. As explained in Refs. [18], [36], generalised synchronisation appears due to the existence of a low-dimensional synchronous manifold, often a very complicated and unknown manifold.

An important contribution to understand why generalised synchronisation is a ubiquitous property in complex network is given by the numerical work of Ref. [38] and the theoretical work of Ref. [39]. In Refs. [38], [39] the ideas of Ref. [40] are extended to complex networks. In Ref. [38], it is shown that generalised synchronisation in heterogeneous degree complex networks is behind the appearance of a synchronisation behaviour where hub nodes provides a skeleton about which synchronisation is developed. The work of Ref. [39] shows that generalised synchronisation occurs whenever there is at least one node whose modified dynamics is periodic. The modified dynamics is a set of equations constructed by considering only the variables of the response system. All the nodes that have a stable and periodic modified dynamics become synchronous in the generalised sense with the nodes that have a chaotic modified dynamics. The general theorem presented in Ref. [39] is a powerful tool for the understanding of weak forms of synchronisation or desynchronous behaviours in complex networks. However, identifying the occurrence of generalised synchronisation does not give much information about the behaviour of the network, since the function that relates the trajectory among the nodes that are generalised synchronous is usually unknown. The CAS phenomenon allows one to calculate, at least in an approximate sense, the equations of motion that describes the pattern to which the nodes are locked to. More specifically, we can derive the set of equations governing, in an approximate sense, the time evolution of the nodes, not covered by the theorem in Ref. [39].

Finally, if there is a node whose modified dynamics describes a stable periodic behaviour and its CAS pattern is also a stable periodic stable behaviour, then the CAS phenomenon appears when the network presents generalised synchronisation.

### About the expected value of the local mean field: the Central Limit Theorem

The Theorem states that, given a set of 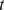 observations, each set of observation containing 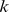 measurements (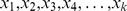), the sum 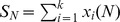 (for 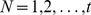), with the variables 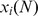 drawn from an independent random process that has a distribution with finite variance 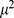 and mean 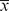, converges to a Normal distribution for sufficiently large 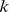. As a consequence, the expected value of these 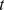 observations is given by the mean 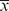 (additionally, 
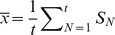
), and the variance of the expected value is given by 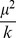. The larger the number 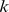 of variables being summed, the larger is the probability with which one has a sum close to the expected value. There are many situations when one can apply this theorem for variables with some sort of correlation [41], as it is the case for variables generated by deterministic chaotic systems with strong mixing properties, for which the decay of correlation is exponentially fast. In other words, a deterministic trajectory that is strongly chaotic behaves as an independent random variable in the long-term. For that reason, the Central Limit Theorem holds for the time average value 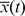 produced by summing up chaotic trajectories from nodes belonging to a network that has nodes weakly connected. Consequently, the distribution of 
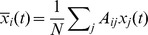
 for node 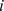 should converge to a Gaussian distribution centred at 
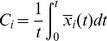
 as the degree of the node is sufficiently large. In addition, the variance 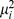 of the local mean field 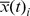 decreases proportional to 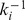, as we have numerically verified for networks of Hindmarsh-Rose neurons (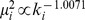) and networks of Kuramoto oscillators (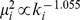).

If the network has no positive Lyapunov exponents, we still expect to find an approximately constant local mean field at a node 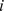, as long as the nodes are weakly connected and its degree is sufficiently large. To understand why, imagine that every node in the network stays close to a CAS pattern and one of its coordinates is described by 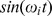. Without loss of generality we can make that every node has the same frequency 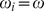. The time-lag property in the node trajectories, when they exhibit the CAS pattern, results in that every node is close to 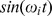 but they will have a random time-lag in relation to the CAS pattern (due to the decorrelated property between the node trajectories). So, the selected coordinate can be described by 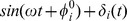, where 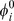 is a random initial phase and 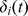 is a small random term describing the distance between the node trajectory and the CAS pattern. Neglecting the term 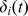, the distribution of the sum 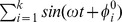 converges to a normal distribution with a variance that depends on the variance of 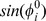.

From previous considerations, if the degree of some of the nodes tend to infinite, the variance of the local mean field for those nodes tends to zero and, in this limit, the residual term 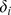 in Eq. (5) is zero and the local mean field of these nodes is a constant. As a consequence, the node is perfectly locked with the CAS pattern (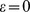 in Eq. (1)).

### Preserving the CAS pattern in different networks: a way to predict the onset of the CAS phenomenon in larger networks

Consider two networks, 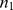 and 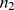, whose nodes have equal dynamical descriptions, the network 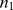 with 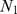 nodes and the network 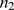 with 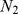 nodes (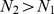), and two nodes, 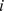 in the network 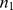 and 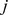 in the network 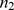. Furthermore, assume that both nodes have stable periodic CAS patterns (**criteria 1** is satisfied), and assume that the nodes have sufficiently large degrees such that the local mean field of node 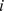 is approximately equal to node 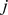. Then the CAS pattern of node 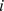 will be approximately the same as the one of node 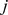 if
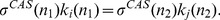
(19)

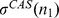
 and 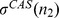 represent the largest coupling strengths for which the variance of the local mean field of a node decays with the inverse of the degree of the node (**criterion 2** is satisfied) in the networks, respectively, and 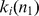 and 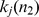 are the degrees of the nodes 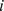 and 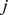, respectively. In other words, the CAS phenomenon occur in the network if 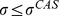.

Therefore, if 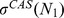 is known, 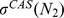 can be calculated from Eq. (19). In other words, if the CAS phenomenon is observed at node 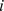 for 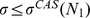, the CAS phenomenon will also be observed at node 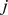 for 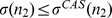, where 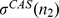 satisfies Eq. (19).

### Conclusions

Concluding, in this work we introduce the phenomenon of Collective Almost Synchronisation (CAS), a phenomenon that is characterised by having nodes possessing approximately constant local mean fields. The appearance of an approximately constant mean field is a consequence of a regime of weak interaction between the nodes responsible to place the node trajectory around stable periodic orbits.

The larger is the degree (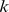) of a node, the higher is the probability that the local mean field is close to an expected value and the smaller the variance (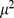) of the local mean field. In fact, the CAS phenomenon appears if 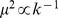, meaning that the Central Limit Theorem is verified for nodes that present the CAS phenomenon. Despite this fact, nodes that present the CAS phenomenon will also be almost, time-lag, phase, and generalised synchronised (see Supplementary information for conditions when generalised synchronisation appears). A peculiar characteristic of networks that present this phenomenon is that nodes can behave in an infinitely large number of different ways, being that the coupling strength among them is very small. If the brain creates memory by minimising the coupling strength and by maximising the number of patterns, this phenomenon might be a possible explanation for the way the brain manages and forms short-term and long-term memory.

A network has the CAS phenomenon if the Central Limit Theorem can be applied and it exists an approximately constant mean field. Alteration in parameters might change the absolute value of the expected value of the local mean field. However, if the Central limit Theorem can still be applied, changes in parameters are not able to destroy the presence in the network of either the CAS phenomenon or weak forms of synchronisation among the nodes.
